# HPV16 synthetic long peptide (HPV16-SLP) vaccination therapy of patients with advanced or recurrent HPV16-induced gynecological carcinoma, a phase II trial

**DOI:** 10.1186/1479-5876-11-88

**Published:** 2013-04-04

**Authors:** Mariette I E van Poelgeest, Marij J P Welters, Edith M G van Esch, Linda F M Stynenbosch, Gijs Kerpershoek, Els L van Persijn van Meerten, Muriel van den Hende, Margriet J G Löwik, Dorien M A Berends-van der Meer, Lorraine M Fathers, A Rob P M Valentijn, Jaap Oostendorp, Gert Jan Fleuren, Cornelis J M Melief, Gemma G Kenter, Sjoerd H van der Burg

**Affiliations:** 1 Department of Gynaecology, Leiden University Medical Center, Leiden, The Netherlands; 2 Department of Clinical Oncology, Leiden University Medical Center, Building 1, K1-P, PO box 9600, 2300 RC Leiden, The Netherlands; 3 Department of Radiology, Leiden University Medical Center, Leiden, The Netherlands; 4 Department of Clinical Pharmacy and Toxicology, Leiden University Medical Center, Leiden, The Netherlands; 5 Department of Pathology, Leiden University Medical Center, Leiden, The Netherlands; 6 Department of Immunohematology and Blood Transfusion, Leiden University Medical Center, Leiden, The Netherlands; 7 ISA Pharmaceuticals B.V., Leiden, The Netherlands; 8 Center of Gynaecologic Oncology Amsterdam, Amsterdam, The Netherlands

**Keywords:** Therapeutic vaccine, Synthetic long peptides, Cervical cancer, Immunomonitoring, Immunotherapy, HPV, Survival

## Abstract

**Background:**

Human papilloma virus type 16 (HPV16)-induced gynecological cancers, in particular cervical cancers, are found in many women worldwide. The HPV16 encoded oncoproteins E6 and E7 are tumor-specific targets for the adaptive immune system permitting the development of an HPV16-synthetic long peptide (SLP) vaccine with an excellent treatment profile in animal models. Here, we determined the toxicity, safety, immunogenicity and efficacy of the HPV16 SLP vaccine in patients with advanced or recurrent HPV16-induced gynecological carcinoma.

**Methods:**

Patients with HPV16-positive advanced or recurrent gynecological carcinoma (n = 20) were subcutaneously vaccinated with an HPV16-SLP vaccine consisting of a mix of 13 HPV16 E6 and HPV16 E7 overlapping long peptides in Montanide ISA-51 adjuvant. The primary endpoints were safety, toxicity and tumor regression as determined by RECIST. In addition, the vaccine-induced T-cell response was assessed by proliferation and associated cytokine production as well as IFNγ-ELISPOT.

**Results:**

No systemic toxicity beyond CTCAE grade II was observed. In a few patients transient flu-like symptoms were observed. In 9 out of 16 tested patients vaccine-induced HPV16-specific proliferative responses were detected which were associated with the production of IFNγ, TNFα, IL-5 and/or IL-10. ELISPOT analysis revealed a vaccine-induced immune response in 11 of the 13 tested patients. The capacity to respond to the vaccine was positively correlated to the patient’s immune status as reflected by their response to common recall antigens at the start of the trial. Median survival was 12.6 ± 9.1 months. No regression of tumors was observed among the 12 evaluable patients. Nineteen patients died of progressive disease.

**Conclusions:**

The HPV16-SLP vaccine was well tolerated and induced a broad IFNγ-associated T-cell response in patients with advanced or recurrent HPV16-induced gynecological carcinoma but neither induced tumor regression nor prevented progressive disease. We, therefore, plan to use this vaccine in combination with chemotherapy and immunomodulation.

## Background

The causal role of Human Papillomavirus (HPV) infections in the development of gynecological cancers, in particular cervical intraepithelial neoplasia and cervical carcinoma, has been unambiguously established. Whereas at an early-stage cervical cancer has a low risk of recurrence after treatment (15%), the more advanced cancers (FIGO stages IIB/III/IV) display a risk of up to 70% for recurrence [1-4]. Genital infections with high-risk HPV are very common and the virus is mainly acquired through sexual activity [5-7]. Genital HPV infection is highly prevalent in young sexually active individuals. In the majority of infected subjects the infection is cleared within one year [8,9]. However, infection with the high-risk HPV type 16 (HPV16) is associated with a greater risk for disease progression and HPV16 is the most common type in patients with invasive cervical cancer [10,11]. HPV16 encodes the two tumor-specific oncoproteins E6 and E7 that can elicit a favorable immune response, in which virus-specific interferon-γ (IFNγ)-producing CD4+ cells and CD8+ cytotoxic T-lymphocytes (CTL) are able to control and eliminate virus-infected cells [12,13]. However, in case of an uncontrolled persistent infection with a high-risk type HPV, the expression of the viral oncoproteins E6 and E7 contributes to the development of cervical (pre)malignancies. Apparently, the immune system fails to respond adequately in these patients and this correlates with the absence or weak expansion or activation of HPV16-specific CD4+ and CD8+ T cells in such patients [14-18]. Importantly, the presence of HPV16-specific responses, albeit weak, is associated with prolonged survival in patients with deeply invading tumors [19].

The treatment of advanced cervical cancer consists of platinum-based chemotherapy but, with a response rate of 20% to 30%, is seldom curative and should be considered as palliative treatment. This is reflected in a poor median survival time in these patients; fewer than 20% survive one year [20-22]. Attempts to improve the treatment-strategy by the use of several chemotherapeutics simultaneously had sporadically resulted in higher response rates and a short increase in overall survival [20,23]. A recent phase III trial of four cisplatin-containing doublet combinations in patients with a stage IVB, recurrent or persistent cervical carcinoma showed no statistically significant differences in survival, albeit that a trend in response rate favored the use of cisplatin and paclitaxel [24]. Clearly, there is a great medical need to identify novel treatment strategies for patients with cervical cancer, particularly for patients in the higher risk categories (*e.g.* patients classified as stage IIB or higher).

Recently, we developed a highly immunogenic synthetic long peptide (SLP) vaccine, consisting of long overlapping peptides of the E6 and E7 oncogenic proteins of HPV16 with an excellent treatment profile in animal models [25-28]. Clinical testing of this vaccine in patients with cervical cancer showed that it harbored the capacity to elicit strong and broad HPV16-specific CD4^+^ and CD8^+^ T-cell responses in most of the patients. Furthermore, it revealed that the vaccine’s toxicity was not beyond grade 2 and well tolerated by the patients [29,30]. Treatment of patients with HPV16-positive high-grade vulvar intraepithelial neoplasia (VIN3) with this vaccine resulted in clinical responses including complete regressions [31]. Notably, clinical outcome was correlated with the strength of the vaccine-induced HPV16-specific T-cell response [32]. We here report the results of our study in which the HPV16-SLP vaccine was tested not only for its safety and tolerability but also for its capacity to induce HPV16-specific T-cell responses and clinical responses in patients with advanced or recurrent HPV16-induced gynecological carcinoma.

## Methods

### Patients and vaccination

This was a phase II trial with the objective to determine the immunological and clinical response to immunotherapy with long peptides derived from the HPV16 E6 and E7 protein in patients with a HPV16-induced advanced or recurrent gynecological carcinoma as well as to assess the safety and tolerability of this type of vaccination. The study was approved by the medical ethical committee of the Leiden University Medical Center (P05.086).

### Vaccine and treatment scheme

The vaccine consisted of a mix of 13 overlapping 25-35-mer peptides representing the entire sequence of the E6 and E7 proteins of HPV16 (HPV16-SLP) dissolved in dimethylsulfoxide (DMSO) and admixed with 20 mM phosphate buffer (pH 7.5) and the adjuvant Montanide ISA-51. The vaccine was produced at the GMP facility of the Leiden University Medical Center (LUMC) [29,31]. The vaccine has been administered at a dose of 300 μg per peptide by subcutaneous injection. Vaccinations were carried out maximally 4 times, at different sites, with a 3-weeks interval. All vaccinations were administered to the patients at the LUMC.

### Eligibility criteria

Eligibility requires all of the following criteria: a) Mentally competent patients of 18 years and older, b) Clinical and radiological evidence of recurrent gynecological cancer, with measurable lesions (preferably histopathologically confirmed), c) No curative treatment (surgery or radiotherapy) options, d) HPV16 positive tumor, e) Performance status of WHO 1–2 or Karnofsky-score >60, f) Pre-treatment laboratory findings of white blood cells (WBC) > 3,000 × 10 9/l, lymphocytes > 1,000 × 10 9/l, platelets > 100 × 10 9/l, hematocrit > 30%, g) No indication of active infectious disease other than HPV16 (i.e. no HIV and/or HBV infection), h) No history of autoimmune disease or systemic intercurrent disease which might affect immunocompetence, i) No history of a second malignancy except curatively treated low-stage tumors with a histology that can be differentiated from the gynecological cancer type, j) No radiotherapy, chemotherapy or other potentially immunosuppressive therapy administered within 4 weeks prior to immunotherapy, k) Life expectance of more than 6 months.

### Examinations

Prior to each vaccination the patient was subjected to physical examination and evaluated for performance status, weight, Hb, hematocrit, WBC differential platelets, PT and PTT, serum creatinine, Na, K, bilirubine, AF, gamma GT, ASAT, ALAT and LDH. Additionally after second and fourth vaccination a gynecological examination was performed.

Follow up was performed every 3 months during the first two years by history taking, physical and gynecological examination. Imaging techniques (multidetector CT- or MRI scan) were performed 6–8 weeks after the last vaccination or earlier when clinically relevant.

### Definition and measurement of tumor lesions

Tumor load was assessed using Response Evaluation Criteria in Solid Tumors, version 1.0 (RECIST 1.0). Measurable disease was defined as the presence of at least one measurable lesion. If measurable disease was restricted to a solitary lesion, its neoplastic nature was confirmed by cytology/histology. A lesion was considered measurable, if accurate measurement of the longest diameter was ≥10 mm on CT or MRI.

Non-measurable lesions were defined as all other lesions, including small lesions (<10 mm on CT or MRI), bone lesions and cystic lesions, and non-measurable disease, such as leptomeningeal disease, ascites, pleural/pericardial effusion, inflammatory breast disease, lymphangitis cutis/pulmonis.

Target lesions, defined as all measurable lesions up to a maximum of five lesions per organ and 10 lesions in total, representative of all involved organs were identified, recorded and measured at baseline. Target lesions were selected on the basis of their size (lesions with the longest diameter) and their suitability for accurate repeated measurements (either by imaging techniques or clinically). A sum of the longest diameter (LD) for all target lesions was calculated and reported as the baseline sum LD. The baseline sum LD was used as reference to determine an objective tumor response by comparing this to sum LD of target lesions after therapy. Non-target lesions comprise all other lesions (or sites of disease) of which the presence was recorded at baseline and throughout follow-up. All baseline evaluations were performed as closely as possible to the beginning of treatment and never earlier than 4 weeks before the beginning of the treatment.

### Evaluation of tumor response

The primary endpoint of the study was an objective tumor response. The evaluation of responses was based on RECIST 1.0. The overall response covered both the response of target and non-target lesions.

A complete response (CR) was defined as disappearance of all target lesions. A partial response (PR) was defined as at least a 30% decrease in the sum of the LD of target lesions when compared to the baseline sum LD. Progressive disease (PD) was defined as at least a 20% increase in the sum of the LD of target lesions in comparison with the lowest sum of the LD of target lesions (i.e. nadir, best result) or the appearance of one or more new lesions. Stable disease (SD) is defined as neither a sufficient shrinkage to qualify for PR nor sufficient increase to qualify for PD. For the non-target lesions CR indicated disappearance of all non-target lesions. PR/SD indicated persistence of one or more non-target lesion(s). PD is defined as appearance of one or more new lesions and/or unequivocal progression of existing non-target lesions.

All radiological assessments were performed by a single experienced radiologist (ELvPvM).

### Evaluation of safety and tolerability

Adverse events, injection site reactions and clinical laboratory variables were monitored and scored according to the CTCAE version 3. Injection site reactions were defined as swelling, erythema and tenderness. All patients were examined physically before every vaccination and medical history was taken. Vital sign examination was performed after each vaccination and patients were given a diary to mark all events in the first week after each vaccination.

### Evaluation of the HPV16-specific T-cell response to vaccination

In acknowledgement to the “minimal information about T cell assays (MIATA)” framework detailed information is given provided about the sample, assay, data acquisition, data analysis, laboratory environment [33].

The sample: Peripheral blood mononuclear cells (PBMC) before, after the second and after the fourth vaccination were isolated within 6 hours after blood was drawn, using Ficoll density gradient centrifugation and controlled cryopreserved (Cryosolution) in 90% fetal calf serum (PAA laboratories, Pasching, Austria) and 10% dimethylsulphoxide (Sigma, St Louis, MO, USA). Equal aliquots of cells (10^7^/vial) were stored in the vapor phase of the liquid nitrogen vessel until use [34].

The assay, data acquisition and analysis: PBMC were tested for HPV16-specificity by a set of complementary T-cell immune monitoring assays, including lymphocyte proliferation assay (LST), cytokine bead array (CBA) and IFN-γ-ELISPOT in which cells were stimulated with pools of 22 amino acid long peptides, overlapping by 12 amino acids. All tests have previously been described. Positive and vaccine-induced responses were pre-defined [29,32]. Briefly, antigen-specific T-cell responses were determined in each blood sample by a short-time proliferation assay according to Standard Operating Procedure (SOP). Freshly isolated PBMC were incubated in 8-replicate wells in medium with 10% autologous serum in the presence of the indicated antigens. On day 6, supernatant was harvested for cytokine analysis and the cells pulsed overnight with [^3^H]Thymidine. The mean plus 3 times standard deviation (STD) of the 8 medium control wells was used as cut-off value. The stimulation index (SI) was calculated by dividing the mean of tested wells by the mean of the medium control. A positive proliferative response was defined as a SI of ≥3 provided that the counts of ≥6 out of 8-wells were above the cut-off value. The supernatants isolated on day 6 of the proliferation assay were subjected to a Th1/Th2 inflammation cytometric bead array (CBA) kit (BD Biosciences, Erembodegem, Belgium) according to the instructions of the manufacturer. The cut-off value was 20 pg/ml, except for IFNγ, for which this was 100 pg/ml. Antigen-specific cytokine production was positive when above the cut-off value and at least twice the medium control. An interferon-γ (IFNγ) ELISPOT assay was performed if at least one blood sample was available after 2 vaccinations. PBMC isolated before and after 2 and/or 4 vaccinations were tested to quantify the number of IFNγ-producing HPV-specific T cells according to SOP. Spots were counted with a fully automated computer-assisted-video-imaging analysis system (BioSys 5000). Specific spots per 100.000 PBMC were calculated by subtracting the mean number of spots + 2 times the standard deviation (STD) of the medium only control from the mean number of spots in experimental wells. Antigen-specific T-cell frequencies were considered to be increased compared to non-responders when specific T-cell frequencies were ≥ 1/10.000. A vaccine-induced response was defined as at least a 3-fold increase in the response after vaccination when compared to the baseline sample.

The laboratory environment: The T-cell assays were performed in the laboratory of the department of Clinical Oncology (LUMC, Leiden) that operates under research conditions. Standard operating procedure (SOPs), including predefined criteria for positive responses, were applied by trained personnel. This laboratory has participated in all proficiency panels of the CIMT Immunoguiding Program (http://www.cimt.eu/workgroups/cip/), as well as in IFNγ ELISPOT panels of the Cancer Immunotherapy Consortium, which aim is to harmonize the reporting and assays used for T-cell monitoring [33,35].

### Statistical analysis

Comparisons of the strength of the different types of immune responses were made by analyzing the differences between the groups of patients with a lower or equal versus a higher median survival (12.6 months whole group and 8.8 months for cervical cancer patients only) by the non-parametric Mann–Whitney test using GraphPad InStat Software. For each different type of immune assay the strength was defined as the median specific spot count (ELISPOT), SI (LST) or amount of cytokine production (CBA) obtained for all 6 different peptide pools per patient, of all patients in one group. In order to assess whether the responsiveness to MRM at baseline was associated with the response to HPV after vaccination by each patient the Fishers Exact test was used. All reported p-values are 2-sided and have not been adjusted for multiple comparisons. A p-value ≤0.05 was considered to indicate statistical significance. Statistical comparisons of different parameters between the group of vaccinated cervical cancer patients and a matched cohort were performed using IBM SPSS20 statistics. Kaplan-Meier curves were used to illustrate the survival of the two groups. Paired Wilcoxon signed rank test and a Log Rank (Mantel Cox) test were used to determine the differences in survival between the matched control cohort and vaccinated patients.

## Results

### Patients & Vaccinations

Fifty-five patients with advanced or recurrent gynecological carcinoma were screened between May 2006 and April 2010. Thirty-two were HPV16-positive of which 21 could be recruited for this study. The other HPV16-positive patients refused to participate (n = 4), displayed progressive disease (n = 4) or did not fulfill the inclusion criteria (n = 3). Within the enrolled patient group, 2 patients displayed a HPV16-induced vaginal carcinoma (ID 7, 9), 2 patients a HPV16-induced anal carcinoma (ID 10, 21), and 17 patients a HPV16-induced cervical carcinoma. The mean age at inclusion was 46.8 years (STD 9.3; range 30–63). Patient characteristics are listed in Table 1.

**Table 1:** Patient characteristics

**ID**	**Age**			**Primary tumor**				**Recurrence**				
	**Diagnosis**	**Recurrence**	**Inclusion**	**Type**	**FIGO stage**	**Treatment**	**Chemotherapy**	**Region**	**Interval prim-rec (months)**	**Treatment**	**Chemotype**	**Interval Chemo-1st Vac (months)**
1	40	41	41	cervix	IB1	RH/CHRT	Cisplatin	LR	14			12.0
2	49	51	51	cervix	IIB	CHRT	Cisplatin	D	26			33.3
3	43	46	46	cervix	IB1	RH/RT		D	37			
4	34	35	36	cervix	IB2	CHRT/RH	Cisplatin	D	18			17.1
5	30	30	30	cervix	IB1	RH/CHRT	Cisplatin	LR	6			5.3
6	55	56	56	cervix	IIIB	CHRT	UK	D	3			3.9
7	51	52	53	vagina	IVB	CHRT	Carboplatin/Taxol	D	16	RT		19.6
8	34	36	36	cervix	IB1	RH/RT		D	19	CH	Cisplatin/Topotecan	1.8
9	54	56	56	vagina	IIB	CHRT	Cisplatin	D	20	RT + HT		22.9
10	38	45	46	anus		CHRT	UK	LR	79	CHRT	UK	41.9
11	44	46	46	cervix	IIIB	CHRT	Cisplatin	D	6			4.9
12	36	40	41	cervix	IIA	RH/CHRT	Cisplatin	D	40	HT + CH	Carboplatin/Taxol	4.4
13	62	63	63	cervix	IV	RH/CHRT	Cisplatin	D	11			7.9
14	32	32	32	cervix	IIB	CHRT	Cisplatin	D	8			7.5
15	35	36	36	cervix	IB1	SN/CHRT	Cisplatin	LR	4	SUR		4.3
16	54	54	54	cervix	IV							
17	48	49	50	cervix	IIB	SUR		D	9			
18	58	59	59	cervix	IB2	CHRT/RH	Cisplatin	D	11	RT		
19	52	54	54	cervix	IIA	CHRT	Carboplatin/Cisplatin	LR	31			37.4
20	39	40	41	cervix	IB1	RH		D	14	CHRT + CH	Carboplatin/Taxol	2.2
21	46	47	47	anus		CH	Cisplatin/Vinorelbine		4			6.5

LR, locoregional; D, distant metastasis; RH, radical hysterectomy; CH, chemotherapy; CHRT, chemoradiation; RT, radiotherapy; SN, Sentinal Node Procedure; HT, hyperthermia; SUR, surgery; UK, unknown; FIGO, International Federation of Gynecologists and Obstetricians. FIGO stage IB1 (clinical lesion ≤ 4 cm) and IB2 (clinical lesion > 4 cm) confined to the cervix. Stage II: tumor extension beyond the cervix, but not the distal 1/3 part of the vagina or the pelvic wall (stage IIA: involvement of the proximal 2/3 part of the vagina, stage IIB: parametrial involvement). Stage IIIB, carcinoma has extended to the pelvic wall and/or the distal 1/3 part of the vagina, and/or causes hydronefrosis. Stage IV, carcinomas spread to the mucosa of the bladder or rectum (stage IV A) or distant organs (stage IVB). The interval between the primary tumor and recurrence (prim-rec) is given in months. The interval between the chemotherapy and the 1^st ^vaccination (Chemo-1st Vac) was calculated by using the starting date of the last given chemotherapy and the date of first vaccination as reference points.

Of the 21 included patients, 1 patient died of progressive disease before start of the vaccinations (ID 18). Eleven patients completed all 4 vaccinations (ID 1, 8–11, 13, 14, 17, 19–21). Five patients received three vaccinations (ID 2–4, 12, 15), and 4 patients received 2 vaccinations (ID 5–7 and 16; Table 2).

**Table 2:** Patient vaccination and outcome

**ID**	**Vaccination**				**Clinical outcome**		**Radiology results**				
	**(n) injections**	**Additional treatment**	**Chemotherapeutic drug**	**Interval 1st Vac-Chemo (months)**	**Status**	**Survival (months)**	**Pre-post scans interval (months)**	**Baseline sum LD (mm)**	**Post vaccination sum LD (mm)**	**Target lesions**	**Non-target lesions (RECIST)**
1	4				died	9	8.1	120	161	PD	PD
2	3	RT			died	21	6.4	52	70	PD	PD
3	3	CH + RT	Cisplatin/Topotecan	1.9	died	19	2.4	132	138	SD	PD
4	3				died	8	4.8	149	189	PD	PD
5	2				died	6	1.8	68	89	PD	PD
6	2				died	4					
7	2	CH	Carboplatin/Gemcitabin	1.0	died	13	6.2	NE	NE	NE	NE
8	4	CHRT/SUR	Carboplatin/Taxol	4.2	died	26	5.5	31	61	PD	PD
9	4	CH	Cisplatin/Topotecan	4.0	died	26	3.9	67	67	SD	PD
10	4				died	25	4.7	68	88	PD	PD
11	4				died	7					
12	3				died	15					
13	4				died	5	3.6	87	80	SD	PD
14	4				died	7	1.3	112	151	PD	PD
15	3				died	8	3.5	0	0		PD
16	2				died	7					
17	4	CH	Cisplatin/Topotecan	2.1	UK	20					
18	0				died	4					
19	4				died	15					
20	4				died	37					
21	4				died	12	4.7	63	79	PD	PD

CH, chemotherapy; CHRT, chemoradiation; LD, longest diameter; NE, not evaluable; PD, progressive disease; RT, radiotherapy; SD, stable disease, SUR, surgery. The interval between the 1^st ^vaccination and the chemotherapy after the vaccinations (1st Vac-Chemo) was calculated by using the date of first vaccination and the starting date of the given chemotherapy during/after the vaccinations as reference points.

### Safety and tolerability

Tumor progression was the main reason for early termination in the study. All patient deaths occurred due to progressive disease. One patient stopped after 3 vaccinations because of persisting flu-like symptoms and swelling of vaccination sites (ID 2). Overall, the vaccine was well tolerated. None of the systemic and local adverse events exceeded CTCAE grade 2 (Table 3). The vaccination was accompanied mostly with erythema and swelling of the skin (Table 4). Hematological values assessed in the blood samples drawn before and after vaccination did not show significant changes (Wilcoxon signed rank test; not shown).

**Table 3:** Systemic and local adverse events in 20 patients who received at least one vaccination

	**Patients (N = 20)**									**Vaccinations (N = 67)**								
	**CTCAE grade 1**			**CTCAE grade 2**			**CTCAE grade 3**			**CTCAE grade 1**			**CTCAE grade 2**			**CTCAE grade 3**		
	**<24 h**	**>24 h**	**total**	**<24 h**	**>24 h**	**total**	**<24 h**	**>24 h**	**total**	**<24 h**	**>24 h**	**total**	**<24 h**	**>24 h**	**total**	**<24 h**	**>24 h**	**total**
**Systemic adverse events**																		
Fever	6 (30%)	1 (5%)	5 (25%)	2 (10%)	1 (5%)	3 (15%)	-	-	-	7 (10.5%)	1 (1.5%)	7 (10.5%)	3 (4.5%)	1 (1.5%)	4 (6%)	-	**-**	**-**
Chills/rigors	6 (30%)	1 (5%)	6 (30%)	-	-	-	-	-	-	10 (15%)	1 (1.5%)	11 (16.5%)	-	-	-	-	**-**	**-**
Myalgia (yes/no)	0 (0%)	1 (5%)	1 (5%)	-	-	-	-	-	-	0 (0%)	1 (1.5%)	1 (1.5%)	-	-	-	-	**-**	**-**
Fatigue	0 (0%)	1 (5%)	1 (5%)	2 (10%)	3 (15%)	3 (15%)	-	-	-	1 (1.5%)	4 (6%)	3 (4.5%)	4 (6%)	4 (6%)	6 (9%)	-	**-**	**-**
Nausea	6 (30%)	2 (10%)	6 (30%)	-	-	-	-	-	-	7 (10.5%)	3 (4.5%)	9 (13.5%)	-	-	-	-	**-**	**-**
Vomiting	2 (10%)	1 (5%)	2 (10%)	-	-	-	-	-	-	2 (3%)	2 (3%)	3 (4.5%)	-	-	-	-	**-**	**-**
Headache	-	-	-	1 (5%)	2 (10%)	2 (10%)	-	-	-	1 (1.5%)	1 (1.5%)	1 (1.5%)	2 (3%)	3 (4.5%)	3 (4.5%)	-	**-**	**-**
Rash/Generalised erythema	3 (15%)	1 (5%)	3 (15%)	1 (5%)	-	1 (4%)	-	-	-	5 (7.5%)	1 (1.5)	5 (7.5%)	1 (1.5%)	-	1 (1.5%)	-	**-**	**-**
Inability to concentrate (Y/N)	-	-	-	-	-	-	-	-	-	0 (0%)	0 (0%)	0 (0%)	-	-	-	-	**-**	**-**
Tingling extremities	1 (5%)	2 (10%)	2 (10%)	-	-	-	-	-	-	1 (1.5%)	0 (0%)	1 (1.5%)	-	-	-	-	**-**	**-**
Swelling extremities	2 (10%)	2 (10%)	4 (20%)	-	-	-	-	-	-	3 (4.5%)	2 (3%)	5 (7.5%)	-	-	-	-	**-**	**-**
Flu-like symptoms/Malaise	4 (20%)	3 (15%)	7 (35%)	-	-	-	-	-	-	5 (7.5%)	5 (7.5%)	10 (15%)	-	-	-	-	**-**	**-**
**Local adverse events**																		
Injection site reaction			0 (0%)			20 (100%)			0 (0%)			0 (0%)			67 (100%)			0(0%)

The number of patients (and percentage) are depicted per catagory (systemically or locally) and grade of toxicity according to CTCAE version 3.

**Table 4:** Injection site reactions in 20 patients who received at least one vaccination

**Injection sites**	**Number (%)**
**Swelling**	
<4	0 (0%)
4-8	13 (65%)
>8	7 (35%)
**Erythema**	
mild	4 (20%)
moderate	16 (80%)
severe	0 (0%)
**Temp**	
mild	4 (20%)
moderate	15 (75%)
severe	1 (5%)
**Pain**	
mild	17 (85%)
moderate	3 (15%)
severe	0 (0%)
**Itching**	
mild	17 (85%)
moderate	3 (15%)
severe	0 (0%)
**Ulceration**	0 (0%)

All the injection sites were scored 1 hour after administration of the vaccine. The pain and itching is scored at the following visit (i.e. 3 weeks after the vaccination by using the diary). Only the maximal injection site reaction per patient is scored.

### Tumor response and survival

The last follow-up was performed in December 2011. In 11 of the 20 vaccinated patients target lesions were identified and measured before and after vaccination (Table 2). In 8 patients post-vaccination scans were not made because of refusal to undergo radiological examinations (ID 20) or because of clinically progressive disease or death. In 3 patients, the target lesions displayed stable disease (SD) while the target lesions of all other 8 patients were classified as progressive disease (PD). In 12 of the vaccinated patients non-target lesions could be evaluated. The overall tumor response in all 12 patients in whom a response could be assessed, was progressive disease.

After immunotherapy, 3 patients were treated with chemotherapy only, 2 patient with chemo-radiation therapy and one patient with radiotherapy only. In December 2011, the median overall survival of the vaccinated group (n = 20) was 12.6 months (STD 9.1; range 4–26 months) after diagnosis of recurrence and 27.3 months (STD 23.8; range 7–56 months) after primary diagnosis. Recalculation of the survival for the 16 vaccinated patients with HPV16-induced cervical carcinoma revealed a median overall survival of 8.8 months (STD 9.2; range 4–37 months) after diagnosis of recurrence and 24.5 months (STD 18.2; range 7–56) after primary diagnosis. One patient (ID 20) was alive in a clinically good condition at 19.4 months after 4 vaccinations (December 2011), she came back with progressive disease in May 2012 but was still alive in October 2012.

### Immune responses

**Figure 1:**
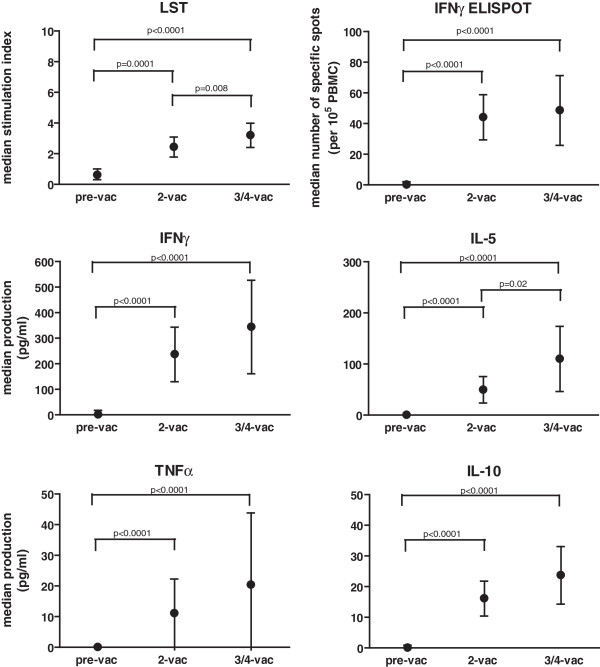
**Vaccination results in stronger HPV16-specific immune responses. **The strength (median + interquartile range) of the indicated immune response to all 6 pools of HPV16 E6 and E7 peptides for the whole group measured before vaccination (pre-vac), after 2 vaccinations (2-vac) and after the 3^rd ^or 4^th^ vaccination (3/4-vac) is given. Only when the strength of the immune response was significantly different, this is indicated by the p-value. Measured was the vaccine-induced proliferation as indicated by the stimulation index using the lymphocyte stimulation test, the antigen-specific increase in the numbers of IFNγ-producing T cells by ELISPOT, and the antigen-specific production of cytokines (IFNγ, IL-5, TNFα and IL-10) in the supernatant of the lymphocyte stimulation test detected by cytokine bead array.

Blood samples were isolated from 20 patients before vaccination and from 15 patients after the second vaccination, from 11 of whom blood was also isolated after either the third or fourth vaccination. In 9 patients a vaccine-induced HPV16-specific proliferative response after the second and/or last vaccination was found. A significant increase in the strength of proliferation was noted after either 2 vaccinations and after 3 to 4 vaccinations (p < 0.0001, Mann Whitney) compared to baseline values. Furthermore, the strength of the response increased between 2 vaccine doses and the last vaccination (p = 0.008, Mann Whitney) (Figure 1). Detailed data is provided in an additional table (Additional file 1). No significant differences were found in the strength of the response to the mix of common recall antigens (MRM) when the reactivity between the pre-vaccination blood sample and that of the second or last vaccination, shown in an additional figure (Additional file 2) were compared, indicating that the increase in HPV16-specific T-cell reactivity was vaccine-mediated.

Analysis of the cytokines produced in the lymphocyte stimulation test (LST) upon stimulation with the HPV16 peptide pools revealed the production of IFNγ in 7 patients (0–4845 pg/ml) and IL-5 in 11 patients (0–1602 pg/ml). Low amounts of TNFα were detected in 6 patients (0–796 pg/ml) and that of IL-10 in 11 patients (0–196 pg/ml). In 4 cases all 4 cytokines were detected (ID 1, 10, 19 and 20), in 1 case (ID21) only IL-5 and IL-10 was detected and in 1 case (ID 8) only IL-10 was detected. There was a significant increase in the level of IFNγ and IL-5 production after the second and after the last vaccination (Figure 1). This was also observed for TNFα and IL-10. The strength of the cytokine response increased between the second and last vaccination, although this was only significant for IL-5 (Figure 1). For none of these cytokines a significant increase in the response to the mix of recall antigens (MRM) after any of the vaccinations was observed (Additional file 2), indicating that the increase in HPV-specific cytokine production was induced by vaccination.

An HPV16-specific vaccine-induced IFNγ-associated immune response as measured by IFNγ-ELISPOT was detected in 12 of the 13 evaluable patients and possibly in 15 patients, as for 3 patients the pre-vaccination sample was not evaluable or not available, shown in an additional table (Additional file 3). There was a significant increase in the strength of the frequency of IFNγ producing T cells as measured by IFNγ-ELISPOT after the second and after the last vaccination (p < 0.0001, Mann Whitney). There was no significant increase in the strength of the response between the second and last vaccination (Figure 1). As expected, no statistical significant differences were found in reactivity to MRM in the samples isolated before and after vaccination (Additional file 2). Of note, the non-statistical significant small increases in cytokine production or number of spots upon stimulation with MRM seen after 3–4 vaccinations probably is due to the underrepresentation of patients who performed poorly as they often did not receive more than 2 vaccinations and responded also less to vaccination (see below). Comparison of the results from the analysis of IFNγ secreted by T cells during proliferation with that of the IFNγ-ELISPOT assay showed no discrepancies with respect to the detection of positive responses, albeit that the IFNγ-ELISPOT assay detected HPV-specific IFNγ production in an additional 5 cases (ID 8, 12, 15, 17, 21).

We then assessed whether the capacity of the patients to respond to the HPV16-SLP vaccine was associated with their general immune status at the start of the trial. Analysis of the data set of 16 patients tested for HPV16-specific reactivity by LST and/or IFNγ-ELISPOT revealed that of the 6 patients who lacked a MRM-specific immune response at the start, 3 responded to the vaccine as detected by either one of the two assays whereas 3 did not. All of the 10 patients who displayed MRM-specific reactivity at the start of the trial also mounted an HPV-specific response after vaccination (p = 0.04) as detected by either one of the two assays. A similar observation was made when the responsiveness as measured by proliferation was analyzed, albeit that this was not significant (p = 0.06).

### Comparison of overall survival with HPV-specific T-cell reactivity

**Figure 2:**
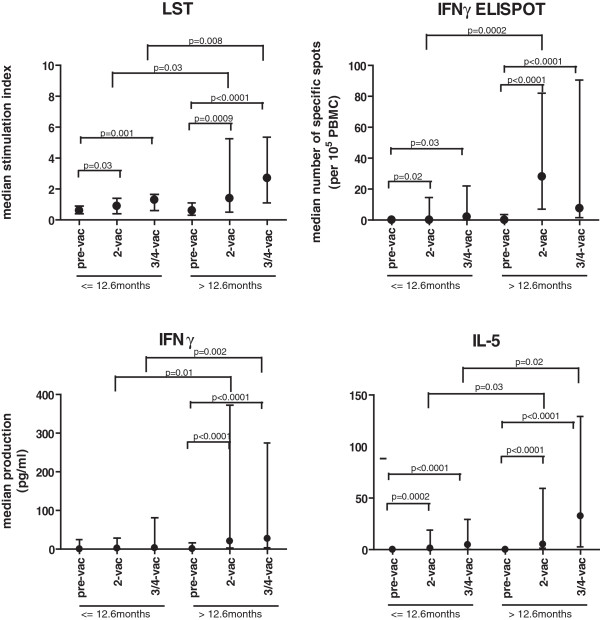
**The group of relatively longer living patients displays a stronger HPV16-specific immune response upon vaccination. **The patients are grouped according to the median survival time (12.6 months) of the whole group. The strength (median + interquartile range) of the indicated immune response to all 6 pools of HPV16 E6 and E7 peptides for the group of patients with survival time equal or less than 12.6 months versus that of the group of patients with a survival time beyond 12.6 months measured before vaccination (pre-vac), after 2 vaccinations (2-vac) and after the 3^rd ^or 4^th ^vaccination (3/4-vac) is given. Only when the strength of the immune response was significantly different, this is indicated by the p-value.

The median survival of this group of patients (n = 20) was 12.6 months. Therefore, the group of patients was divided into a cohort of patients with an overall survival of 12.6 months or less and a cohort of patients surviving longer than 12.6 months. Both groups of patients showed a significant increase in the strength of HPV16-specific proliferation or number of HPV16-specific T cells as measured by IFNγ-ELISPOT after the second and after the last vaccination (Figure 2). However, the patients that lived longer displayed a significantly stronger immune response after the second and/or the last vaccination than the patients with a relative short survival, and this was reflected in all assays (Figure 2). Detailed data is provided in an additional table (Additional file 4). There was no significant difference in HPV-specific reactivity between the groups before they were vaccinated nor was there a difference between the groups in their reactivity to MRM at all time points tested.

We then assessed the relationship between HPV16-specific immune reactivity and the survival within the group of patients with a cervical carcinoma only. The median survival was 8.8 months and the patients were divided into two groups accordingly (Additional file 5). Not enough patients were tested by IFNγ-ELISPOT so that the analysis was limited to the results of the LST and associated cytokine production. The HPV-16-specific proliferation was weak and on average below the cut-off of the proliferation assay (SI >3) for the group of patient with a short survival while the group of patients surviving longer on average displayed an HPV16-specific proliferation above this cut-off value after the vaccinations (Additional file 5). When compared to the group of patients with a relatively short survival, the strength of proliferation was higher for the group of longer survivors after the second (median SI = 0.6 *vs* SI = 1.6; p = 0.0006) and after the last vaccination (median SI = 1.3 *vs* SI = 2.3; p = 0.04). Analysis of the cytokine responses revealed a pattern that reflected the proliferative responses. The strength of IFNγ and IL-5 production was very low in the group with relatively low survival while in the group of longer survivors the median of IFNγ (p < 0.0001) and IL-5 (p < 0.0001) production was higher and increased during the vaccination period (Additional file 5). Because of the low amounts of TNFα and IL-10 produced, these cytokines were not analyzed with respect to survival. Overall, it became clear that the group of patients with cervical cancer who lived relatively longer also displayed a stronger and more functional vaccine-induced HPV16-specific T-cell response.

### Comparison of overall survival with a matched cohort group of cervical cancer patients

**Figure 3:**
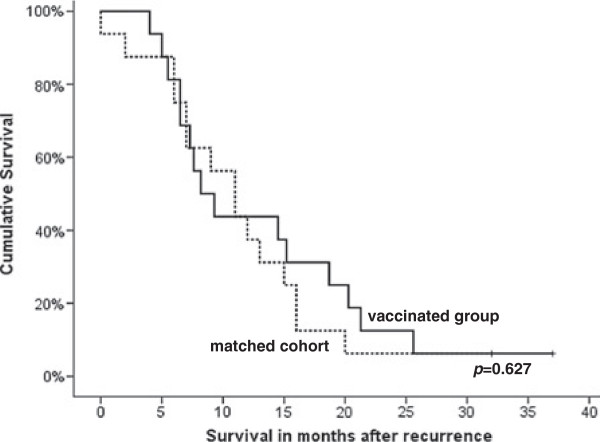
**Comparison of overall survival after cervical cancer recurrence between vaccinated patients and a matched cohort of non-vaccinated cervical cancer patients. **The survival of the group of 16 vaccinated patients with a cervical carcinoma was compared to a cohort group of non-vaccinated patients who where primarily matched for FIGO stage, time to recurrence, primary treatment and salvage therapy after recurrence and which turned out to be matched also for age of diagnosis, age of recurrence, the type of primary and salvage chemotherapy as well as for adjuvant therapy. Both the log-rank and the Wilcoxon signed rank test revealed no difference in survival between the two groups.

Notably, the unique characteristics of immunotherapeutic agents may induce cancer-specific immune responses far before affecting tumor growth. Frequently, there is a delayed detection of clinical activity after immunotherapeutic treatment, and the RECIST criteria may not offer a complete description of the response to immunotherapeutic agents [36]. We therefore constructed an equally sized historical control group of cervical cancer patients, who were all treated within the LUMC and who were matched with the vaccinated patients for a number of clinical parameters in the following order: FIGO stage, time to recurrence, primary treatment and salvage therapy for recurrence (Additional file 6). The matched cohort group displayed no differences in age of diagnosis, age of recurrence, the type of primary and salvage chemotherapy or the type of adjuvant therapy but did differ with respect to the site of recurrence (Additional file 6). The loco-regional recurrences were overrepresented in the matched cohort and this is known to be associated with better survival. Indeed evaluation of the survival after recurrence revealed a median survival of 8.5±9.4 months of the vaccinated group and 11.0±7.7 months of the matched cohort group but survival was not significantly different (p = 0.59, Wilcoxon signed rank test). The use of the 5-year Kaplan-Meier method and the log-rank test in order to determine differences in overall survival showed no significant differences (p = 0.63, log rank test; Figure 3 and Additional file 6).

## Discussion

The capacity of the HPV16-SLP vaccine to induce HPV16-specific T-cell responses was tested in 20 patients with advanced or recurrent gynecological cancer. Similar to our previous studies [29-32] immunotherapy with synthetic long peptides representing the sequence of the oncogenic proteins E6 and E7 of high risk HPV16 admixed with Montanide ISA-51 adjuvant was safe and able to induce HPV16-specific T-cell responses. Most of the toxicities seen did not go beyond grade II and consisted of discomfort and low grade fever in the first 48 hours after injection. Swelling at the vaccination sites was often graded as II. In 13 of the 16 patients who could be evaluated immunologically an HPV16-specific T-cell response was induced after 2 to 4 vaccinations. Patients who failed to mount an HPV16-specific T-cell response also lacked robust immunity against common recall antigens, suggesting that they may have had a generally disease-induced decreased T cell immunocompetence,

A vaccine-induced immune response did not result in clear regressions of the tumor according to RECIST. Division of the patients based on the median survival showed that the group of patients who did relatively well also mounted a stronger immune response upon vaccination, although the vaccine-induced HPV16-specific immune response was not as strong as reported for vaccinated patients with pre-malignant VIN [31,32]. This was also found when all patients with non-cervical carcinomas were excluded. It is unclear whether the correlation between survival time and strength of the immune response to HPV16 vaccination reflects a better overall immune reactivity of patients who live longer or whether a somewhat better vaccine-induced immune response to HPV16 resulted in a longer survival of these patients. Notably, of the 10 patients with a survival beyond the median survival time of 12.6 months, 9 had received additional therapy after recurrence or vaccine therapy, whereas in the group of 10 patients with a relatively lower survival time only 2 had received additional therapy after recurrence indicating that the influence of additional conventional therapy should also be taken into account as a factor for better survival. In order to gain better insight in the potential impact of vaccination on the survival of patients with an advanced stage or recurrent cervical carcinoma we constructed a control cohort. The mean and median survival time after recurrence did not differ with that of the vaccinated cohort suggesting that the increased strength of the vaccine-induced HPV16-specific T-cell response observed in the group of patients with a relatively longer survival most likely reflects the overall fitness of these patients but does not add to the survival of these patients.

Previously, a group of 43 patients with end-stage cervical cancer had been vaccinated with this HPV16 synthetic long peptide vaccine, one of whom showed a complete response after vaccination and 5 of whom showed stable disease [30]. The patient with a complete response was treated with chemo-radiation before vaccination, whereas 4 of the 5 cases with stable disease received chemotherapy after vaccination. Among the long survivors (>12.6 months) with cervical cancer (n = 8) in the current trial, 3 had received chemotherapy prior to vaccination and 3 received chemotherapy after vaccination. This suggests that there is no overt relation between the timing of standard therapy, before or after immunotherapy, and clinical outcome. In addition, there was no relation between the timing of chemotherapy, before or after immunotherapy, and the strength of the measured immune responses. In most of the patients chemotherapy was given months before the start of immunotherapy. In a few cases chemotherapy was given after they had received 2 or more vaccinations but a negative effect of the different types of platinum-based chemotherapy is not to be expected as it was previously shown that platinum-based therapy did not affect the immunogenicity of DC vaccination [37]. However, the direct and indirect effects of the different types of chemotherapy regimens on the priming and function of T-cell immunity in patients with cervical cancer should be studied in a controlled fashion. Our recent pre-clinical data indicate synergy of HPV long peptide vaccination and chemotherapy in effective therapy of established transplantable HPV16+ tumors in mice (unpublished observations). Furthermore, in a pilot study where patients with advanced cervical carcinoma were given a single HPV16 long peptide vaccine dose properly timed during carboplatin and paclitaxel chemotherapy, revealed that a chemotherapy induced change in myeloid cell populations coincided with a remarkably robust induction of HPV-specific immune responses (unpublished observations). This indicates that the synergy observed in mice may be extended to patients when proper timing of chemotherapy and immunotherapy are taken into account.

## Conclusions

In patients with cervical cancer, several immunotherapeutic strategies have been explored in clinical trials. Therapeutic vaccines employing vector-based, peptide- or protein-based, nucleic acid-based, and cell-based therapeutic vaccines targeting the HPV16 E6 and/or E7 antigens recombinant viral vectors, have been tested in attempts to increase the magnitude and quality of the HPV16-specific immune responses to treat HPV16-driven cervical cancer (reviewed in [18,28]). While we here can conclude that the induction of HPV16-specific immunity in patients with advanced or recurrent cervical cancer patients is feasible we did not see a hint that may indicate that this vaccine regimen may bear clinical impact. Whereas the first results of vaccinating patients with HPV16-induced premalignant disease are clinically promising, it is clear that clinical improvement by vaccination only is not likely to happen in patients with advanced or recurrent cervical cancer probably because of a large tumor burden and associated local immune suppression which can hamper T cells to exert their full effector function However, real advances may be expected from combination of therapeutic HPV vaccination with carefully timed standard chemotherapy which also has immunostimulatory properties [38]. Our unpublished data indicate that combined therapy involving the use of carboplatin and paclitaxel may act at least by relief of immune suppression by myeloid cells that are present within carcinoma’s [39-41]. Other attractive options for combination with vaccination are the use of immunemodulating compounds that polarize Th1 reactivity such as pegylated type I interferon. We recently observed that vaccine-induced T cell immunity was strongly improved when vaccination was combined with IFNα in a vaccine trial in colorectal cancer patients [42]. Improved responsiveness may also be achieved by a combination of the vaccine with checkpoint control blocking antibodies such as those blocking PD-1 as PD-1 is expressed by many cervical cancer infiltrating T cells [43] and antibody-mediated blocking of PD-1 displayed clinical success in a number of different immunogenic cancers [44]. In addition, therapies that deplete tumor-specific regulatory T cells which are also present in cervical carcinoma may proof beneficial [16,32,45,46]. It can thus be envisaged that therapeutic efficacy will be reached by combination therapy used in a well coordinated fashion allowing vaccine induced immunity to take control of the tumor.

## Competing interests

This study has been conducted by the Leiden University Medical Center (LUMC), which holds a patent on the use of synthetic long peptides as vaccine (US 7.202.034). CJMM and SHvdB are named as inventors on this patent. The LUMC does not share the financial benefit from this patent with its employees. CJMM has been employed part-time (75%) since January 20, 2008, and full time since January 2012 by ISA Pharmaceuticals, which exploits this long-peptide vaccine patent, and has been granted options on ISA Pharmaceuticals stock. All other authors declare that they have no conflict of interest.

## Authors’ contributions

MIEvP, MJPW, JO, GJF, CJMM, GGK and SHvdB were involved in the conception and design of the study. LMF, ARPV, JO designed, produced and validated the HPV16 SLP vaccine used in this study, MIEvP, EMGvE, GK, MvdH, MJGL, DMABvdM and GGK performed all clinical tasks, acquired, analyzed and interpreted all clinical data. ELvPvM analyzed and interpreted all radiological data obtained, MJWP, LFMS, GJF, CJMM and SHvdB aquired, analyzed and interpreted the immunological and/or virological data. MIEvP, MJPW, EMGvE, JO, GJF CJMM, GGK and SHvdB drafted the manuscript. All authors approved the final manuscript for publication.

## Supplementary Material

Additional file 1 Summary of the HPV16-specific proliferative responses measured by the lymphocyte stimulation assay. Click here for file

Additional file 2 No differences exist in the immune response to recall antigens during and after the vaccination period. Click here for file

Additional file 3 Summary of the HPV16-specific T-cell frequency measured by IFNγ-ELISPOT. Click here for file

Additional file 4 Strength of immune response versus the median survival of all vaccinated patients. Click here for file

Additional file 5 Strength of immune response versus the median survival of vaccinated cervical cancer patients. Click here for file

Additional file 6 Comparison of vaccinated patients with a matched cohort. Click here for file
