# Supplementary material for: HPV16 synthetic long peptide (HPV16-SLP) vaccination therapy of patients with advanced or recurrent HPV16-induced gynecological carcinoma, a phase II trial
Source: J Transl Med. 2013 Apr 4;11:88. doi: 10.1186/1479-5876-11-88 (PMC3623745; doi:10.1186/1479-5876-11-88)
Supplement: Additional file 1 — Summary of the HPV16-specific proliferative responses measured by the lymphocyte stimulation assay. [file 1479-5876-11-88-S1.pdf]

Additional File 1
Summary of the HPV16-specific proliferative responses measured by the lymphocyte stimulation assay.

ID	pre vaccination							2-vaccinations							3 or 4-vaccinations						
	**E6.1**	**E6.2**	**E6.3**	**E6.4**	**E7.1**	**E7.2**	**MRM**	**E6.1**	**E6.2**	**E6.3**	**E6.4**	**E7.1**	**E7.2**	**MRM**	**E6.1**	**E6.2**	**E6.3**	**E6.4**	**E7.1**	**E7.2**	**MRM**
**1**	0,9	1,0	1,0	1,1	1,4	1,0	**7,5**	0,7	0,7	1,3	1,0	0,7	1,2	2,3	1,2	**6,0**	**7,6**	**3,0**	0,6	1,5	2,9
**2**	0,8	1,6	1,7	1,0	**23,3**	**5,5**	**29,3**	**11,6**	**11,4**	**13,5**	**7,7**	10,2	**15,2**	**12,4**	**8,1**	**7,4**	**4,4**	2,4	3,5	**14,6**	2,3
**3**	0,3	0,3	0,3	0,3	0,2	1,2	**7,3**	**3,6**	**3,5**	2,3	**6,6**	0,6	**3,9**	**4,5**	**8,9**	**15,7**	**8,3**	**4,5**	**10,2**	**11,0**	**16,7**
**4**	0,5	0,4	0,4	0,1	0,5	1,3	2,4	0,5	0,6	0,9	0,3	0,3	1,0	1,0							
**5**	0,8	0,7	0,4	0,2	0,3	1,1	0,8														
**6**	0,3	0,3	0,2	0,1	0,1	0,3	0,3														
**7**	0,2	0,5	0,4	0,1	0,3	1,0	**3,8**														
**8**	0,3	0,5	0,2	0,2	0,2	0,7	2,9	0,5	1,3	1,3	0,9	0,2	2,5	1,2	0,9	3,4	0,7	0,7	1,1	1,1	2,9
**9**	0,5	0,5	0,5	0,4	0,8	0,8	1,0	0,2	0,3	0,3	0,8	0,3	0,4	0,6	1,1	**3,0**	**4,5**	**4,7**	1,0	1,4	1,2
**10**	1,1	**3,0**	1,1	1,5	1,1	**3,9**	**15,5**	0,5	1,2	1,5	0,5	2,3	**4,7**	**4,7**	1,3	**3,0**	**5,4**	3,3	2,4	**7,2**	**8,8**
**11**	0,6	0,7	0,7	0,1	0,7	0,6	0,4								0,6	1,6	1,2	0,2	0,4	0,9	0,2
**12**	0,7	1,5	1,0	0,1	0,4	1,4	1,4	0,4	1,0	2,0	0,2	0,2	1,1	1,3							
**13**	0,9	2,2	1,0	0,6	0,8	2,5	2,0	0,2	0,5	0,1	0,1	0,1	0,7	0,4	1,4	2,0	1,1	1,5	1,6	3,3	**3,4**
**14**	0,5	0,6	0,6	0,0	0,4	0,7	1,2	0,9	1,0	**4,1**	0,2	0,2	**3,9**	0,6							
**15**	0,4	0,7	0,3	0,2	0,4	0,6	**3,8**	0,9	1,5	1,1	0,3	0,6	1,6	1,1							
**16**	**5,1**	1,7	0,5	**10,5**	1,0	1,4	**57,8**														
**17**	0,3	0,5	0,3	0,1	0,3	0,6	2,5	0,7	1,0	0,3	0,1	0,2	0,9	1,7	0,3	0,9	0,4	1,9	0,2	0,7	2,0
**18**																					
**19**	0,7	0,9	0,3	0,5	0,9	0,6	3,1	**6,4**	**7,6**	**6,9**	**4,2**	**5,9**	**5,8**	**5,0**	**5,3**	**4,6**	2,2	**4,9**	**8,7**	**6,6**	2,4
**20**	0,7	1,1	0,6	0,3	0,5	1,0	**4,3**	2,8	**5,9**	2,8	2,7	0,6	2,3	**4,1**	1,4	1,1	1,1	1,1	1,3	1,6	2,4
**21**	0,1	0,4	0,1	0,1	0,1	0,8	**4,8**	2,8	**4,1**	2,9	1,3	3,8	**4,8**	**13,6**	0,6	1,5	0,4	0,4	0,4	1,7	3,1

Indicated is the stimulation index (SI), which was calculated by dividing the mean of tested wells by the mean of the medium control. Indicated in bold are positive proliferative responses defined as a SI of ≥3 provided that the counts of ≥6 out of 8-wells were above the cut-off value (mean plus 3 times standard deviation (STD) of the 8 medium control wells). Fields left open indicate that there was no PBMC sample that could be tested at that time point.
