# Supplementary material for: HPV16 synthetic long peptide (HPV16-SLP) vaccination therapy of patients with advanced or recurrent HPV16-induced gynecological carcinoma, a phase II trial
Source: J Transl Med. 2013 Apr 4;11:88. doi: 10.1186/1479-5876-11-88 (PMC3623745; doi:10.1186/1479-5876-11-88)
Supplement: Additional file 2 — No differences exist in the immune response to recall antigens during and after the vaccination period. [file 1479-5876-11-88-S2.pdf]

Additional File 2

No differences exist in the immune response to recall antigens during and after the vaccination period.

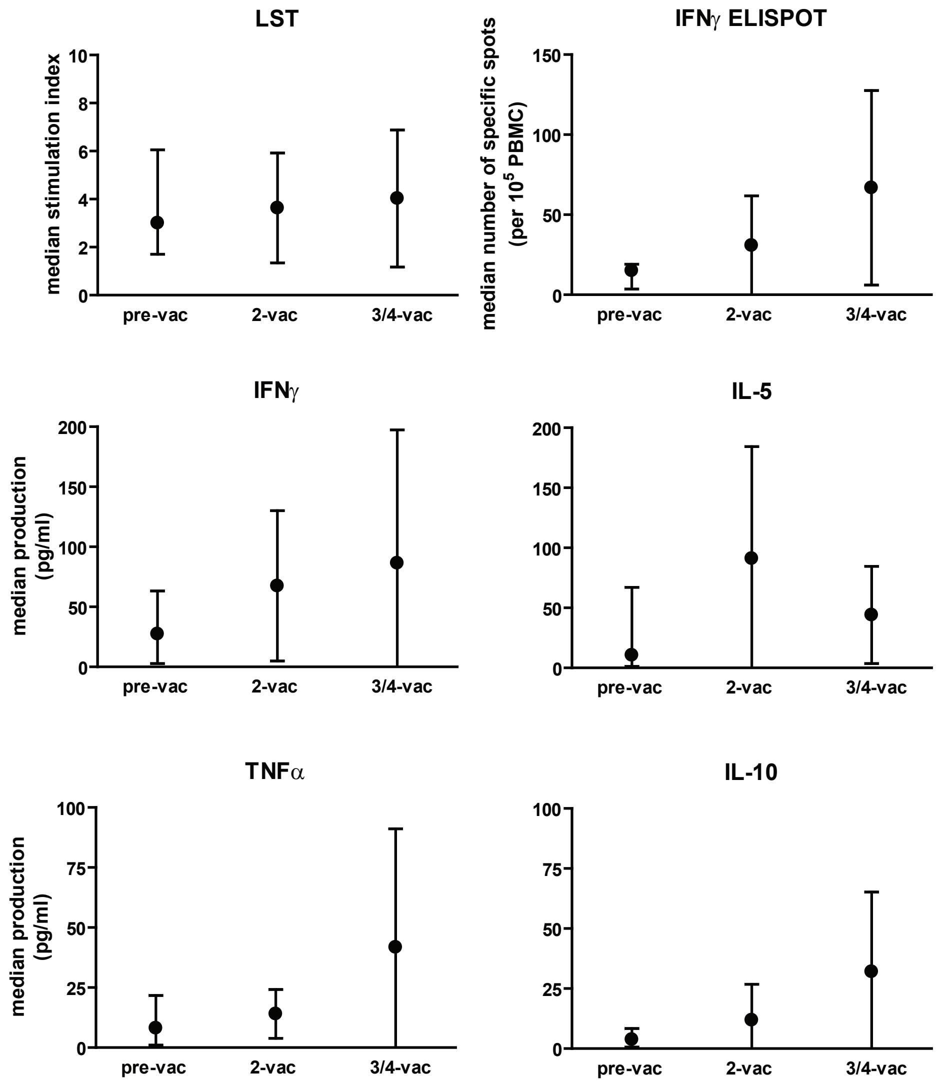

The strength (median+interquartile range) of the indicated immune response against the mix of recall antigens (MRM) for the whole group measured before vaccination (pre-vac), after 2 vaccinations (2-vac) and after the 3rd or 4th vaccination (3/4-vac) is given. Only when the strength of the immune response was significantly different this is indicated by the p-value. None of the immune responses was significantly different at any time point. Measured was the MRM-specific proliferation as indicated by the stimulation index using the lymphocyte stimulation test, the MRM-specific increase in the numbers of IFNγ-producing T cells by ELISPOT, and the MRM-specific production of cytokines (IFNγ, IL-5, TNFα and IL-10) in the supernatant of the lymphocyte stimulation test detected by cytokine bead array.
