# Supplementary material for: HPV16 synthetic long peptide (HPV16-SLP) vaccination therapy of patients with advanced or recurrent HPV16-induced gynecological carcinoma, a phase II trial
Source: J Transl Med. 2013 Apr 4;11:88. doi: 10.1186/1479-5876-11-88 (PMC3623745; doi:10.1186/1479-5876-11-88)
Supplement: Additional file 3 — Summary of the HPV16-specific T-cell frequency measured by IFNγ-ELISPOT. [file 1479-5876-11-88-S3.pdf]

Additional File 3
Summary of the HPV16-specific T-cell frequency measured by IFNγ-ELISPOT.

ID	pre vaccination							2 vaccinations							3 or 4 vaccinations						
	E6.1	E6.2	E6.3	E6.4	E7.1	E7.2	MRM	E6.1	E6.2	E6.3	E6.4	E7.1	E7.2	MRM	E6.1	E6.2	E6.3	E6.4	E7.1	E7.2	MRM
**1**	0	0	0	0	0	0	**11**	**13**	**52**	**32**	0	**15**	3	**50**	**21**	**86**	**94**	8	4	**21**	**76**
**2**	0	0	5	0	**168**	**78**	**163**	**32**	6	6	0	4	**46**	**169**	**60**	**33**	**25**	7	**37**	**126**	**172**
**3**	0	0	4	**40**	0	0	**15**	**23**	**145**	**54**	**93**	0	0	**38**	**213**	**166**	**79**	**102**	**23**	**65**	**147**
**4**								**65**	**78**	**31**	0	0	**27**	9							
**5**																					
**6**																					
**7**																					
**8**	0	8	0	8	0	**12**	0	**18**	**141**	**28**	**17**	**27**	**170**	2	3	3	0	0	4	8	0
**9**															8	**17**	**23**	**52**	0	0	0
**10**	0	0	0	0	1	0	8	0	8	**28**	0	7	**16**	9	0	5	4	3	5	5	3
**11**								0	0	0	0	0	0	0	0	4	**20**	0	0	3	0
**12**	0	3	**12**	5	2	4	**17**	**18**	**82**	**40**	**13**	9	9	**14**							
**13**	0	0	2	0	2	2	4	0	2	0	0	0	6	0	0	5	0	0	0	0	0
**14**	0	0	0	0	0	3	0	0	5	0	0	0	0	0							
**15**	0	0	0	0	0	8	**19**	**17**	**126**	**277**	0	0	**14**	0							
**16**																					
**17**	0	0	0	0	0	3	**19**	7	**72**	2	0	**39**	**63**	2	0	0	0	0	0	0	**19**
**18**																					
**19**	0	0	0	0	**60**	0	**16**	**131**	**205**	**197**	**82**	**118**	**192**	**11**	**190**	**286**	**296**	**12**	**114**	**354**	3
**20**	0	2	0	1	0	0	3	**128**	**246**	**37**	**57**	0	**66**	0							
**21**	0	5	0	0	0	0	**194**	0	**180**	0	0	0	**13**	**105**	0	**132**	0	0	0	**23**	**181**

Indicated is the number of specific spots per 100,000 PBMC, which were calculated by subtracting the mean number of spots + 2xSTD of the medium only control from the mean number of spots in experimental wells. Antigen-specific T-cell frequencies were considered to be increased compared to non-responders when specific T-cell frequencies were above 1/10,000 and are indicated in bold. Fields left open indicate that there was no PBMC sample that could be tested at that time point.
