# Supplementary material for: HPV16 synthetic long peptide (HPV16-SLP) vaccination therapy of patients with advanced or recurrent HPV16-induced gynecological carcinoma, a phase II trial
Source: J Transl Med. 2013 Apr 4;11:88. doi: 10.1186/1479-5876-11-88 (PMC3623745; doi:10.1186/1479-5876-11-88)
Supplement: Additional file 4 — Strength of immune response versus the median survival of all vaccinated patients. [file 1479-5876-11-88-S4.pdf]

Additional File 4
Strength of immune response versus the median survival of all vaccinated patients.

		Survival <= 12.6 months				Survival > 12.6 months		
		**pre-vac**	**2-vac**	**4-vac**		**pre-vac**	**2-vac**	**4-vac**
**ELISPOT**		n=5	n=5	n=3		n=8	n=8	n=6
	Average	0,7	25,2	21,9	Average	8,7	55,8	61,9
	STD	1,7	61,8	39,5	STD	27,8	65,1	96,1
	Median	0,0	0,0	2,0	Median	0,0	28,0	7,5
	IQR25	0,0	0,0	0,0	IQR25	0,0	7,4	2,3
	IQR75	0,0	13,8	21,0	IQR75	3,3	81,6	84,8
		in spots per 100.000 cells				*in spots per 100.000 cells*		
**LST**		n=7	n=6	n=4		n=9	n=9	n=8
	Average	0,7	1,3	1,7	Average	1,3	3,2	3,9
	STD	0,5	1,3	1,8	STD	3,2	3,7	3,7
	Median	0,6	0,9	1,3	Median	0,6	1,4	2,7
	IQR25	0,4	0,4	0,6	IQR25	0,3	0,5	1,1
	IQR75	0,9	1,4	1,6	IQR75	1,1	4,6	5,3
		in stimulation index				*in stimulation index*		
**IFNγ**		n=7	n=6	n=4		n=9	n=9	n=8
	Average	17,9	99,3	183,2	Average	131,8	328,0	424,0
	STD	32,7	214,0	536,2	STD	674,6	620,0	869,1
	Median	0,0	2,2	3,1	Median	0,9	20,7	27,2
	IQR25	0,0	0,0	0,0	IQR25	0,0	3,5	3,4
	IQR75	22,5	27,6	54,7	IQR75	13,2	361,5	261,7
		in pg/ml				*in pg/ml*		
**IL-5**		n=7	n=6	n=4		n=9	n=9	n=8
	Average	1,0	34,4	42,7	Average	3,0	59,5	143,5
	STD	4,6	132,3	133,3	STD	16,0	116,9	314,8
	Median	0,0	1,3	4,7	Median	0,0	5,2	32,5
	IQR25	0,0	0,1	0,3	IQR25	0,0	1,4	2,7
	IQR75	0,3	18,3	27,6	IQR75	0,7	49,7	127,9
		in pg/ml				*in pg/ml*		
**TNFα**		n=7	n=6	n=4		n=9	n=9	n=8
	Average	0,4	1,6	1,6	Average	2,1	17,4	29,7
	STD	0,6	3,3	4,5	STD	9,2	68,6	121,5
	Median	0,1	0,1	0,1	Median	0,0	2,1	2,4
	IQR25	0,0	0,0	0,0	IQR25	0,0	0,2	0,4
	IQR75	0,7	1,7	1,1	IQR75	0,1	6,2	7,2
		in pg/ml				*in pg/ml*		
**IL-10**		n=7	n=6	n=4		n=9	n=9	n=8
	Average	0,5	11,9	13,5	Average	3,7	18,9	28,8
	STD	0,8	24,4	27,6	STD	11,3	28,6	44,3
	Median	0,0	3,3	1,3	Median	0,0	8,9	12,9
	IQR25	0,0	0,0	0,0	IQR25	0,0	0,6	0,0
	IQR75	0,8	13,7	13,2	IQR75	0,9	23,3	36,5
		in pg/ml				*in pg/ml*		

For each assay and time point the number of patients tested and included are indicated. The average, standard deviation (STD), median and the interquartile range with the 25% percentile (IRQ25) and 75% percentile (IRQ75) are provided.
