# Supplementary material for: HPV16 synthetic long peptide (HPV16-SLP) vaccination therapy of patients with advanced or recurrent HPV16-induced gynecological carcinoma, a phase II trial
Source: J Transl Med. 2013 Apr 4;11:88. doi: 10.1186/1479-5876-11-88 (PMC3623745; doi:10.1186/1479-5876-11-88)
Supplement: Additional file 5 — Strength of immune response versus the median survival of vaccinated cervical cancer patients. [file 1479-5876-11-88-S5.pdf]

Additional File 5
Strength of immune response versus the median survival of vaccinated cervical cancer patients.

		Survival <= 8.8 months				Survival > 8.8 months		
		**pre-vac**	**2-vac**	**4-vac**		**pre-vac**	**2-vac**	**4-vac**
**LST**		n=5	n=4	n=2		n=8	n=8	n=6
	Average	0,7	0,9	1,3	Average	1,3	3,4	4,1
	STD	0,5	1,1	0,8	STD	3,3	3,8	4,0
	Median	0,6	0,6	1,3	Median	0,7	1,6	2,3
	IQR25	0,4	0,3	0,8	IQR25	0,3	0,7	1,1
	IQR75	0,7	1,0	1,6	IQR75	1,0	5,8	6,5
		in stimulation index				*in stimulation index*		
**IFNγ**		n=5	n=4	n=2		n=8	n=8	n=6
	Average	7,2	109,1	4,6	Average	152,5	378,0	585,0
	STD	14,5	237,2	8,1	STD	713,8	646,1	952,1
	Median	0,0	1,8	1,6	Median	1,1	52,3	137,4
	IQR25	0,0	0,0	0,0	IQR25	0,0	4,0	14,4
	IQR75	2,0	26,3	4,4	IQR75	30,8	511,7	846,6
		in pg/ml				*in pg/ml*		
**IL-5**		n=5	n=4	n=2		n=8	n=8	n=6
	Average	0,3	7,9	11,4	Average	3,4	66,0	163,9
	STD	0,6	11,3	20,0	STD	17,0	122,5	332,0
	Median	0,0	0,4	0,3	Median	0,0	4,7	40,9
	IQR25	0,0	0,1	0,2	IQR25	0,0	1,4	3,4
	IQR75	0,2	18,3	11,3	IQR75	0,5	70,4	133,3
		in pg/ml				*in pg/ml*		

For each assay and time point the number of patients tested and included are indicated. The average, standard deviation (SD), median and the interquartile range with the 25% percentile (IRQ25) and 75% percentile (IRQ75) are provided.
