# Supplementary material for: HPV16 synthetic long peptide (HPV16-SLP) vaccination therapy of patients with advanced or recurrent HPV16-induced gynecological carcinoma, a phase II trial
Source: J Transl Med. 2013 Apr 4;11:88. doi: 10.1186/1479-5876-11-88 (PMC3623745; doi:10.1186/1479-5876-11-88)
Supplement: Additional file 6 — Comparison of vaccinated patients with a matched cohort. [file 1479-5876-11-88-S6.pdf]

Additional File 6
Comparison of vaccinated patients with a matched cohort

	Vaccine group (n=16)	Mean ± SD (range)	Matched cohort (n=16)	Mean ± SD (range)	Independent Sample T-test for equality of means (p= ; 95% CI)	Significance (p=)
**FIGO**						
1B1	6 (37.5%)		6 (37.5%)			1.0*
1B2	1 (6.2%)		1 (6.2%)			
2A	2 (12.5%)		2 (12.5%)			
2B	3 (18.8%)		3 (18.8%)			
3B	2 (12.5%)		2 (12.5%)			
4	2 (12.5%)		2 (12.5%)			
**Interval diagnosis - recurrence (months)**		15.06 ± 12.2		14.25 ± 9.2	0.83 95% CI (-8.60 - 6.98)	0.833***
**Primary Therapy**						
None	1 (6.2%)		0			0.182*
Surgery	7 (43.8%)		10 (62.5%)			
CHRT	8 (50%)		4 (25%)			
RT	0		2 (12.5%)			
**Recurrence Therapy**						
Vaccination (Vac)	10 (62.5%)		0			**0.002***
CH + Vac	2 (12.5%)		0			
Vac + CH + RT	1 (6.2%)		0			
Vac + RT	1 (6.2%)		0			
CH + Vac + CH	1 (6.2%)		0			
SUR + Vac	1 (6.2%)		0			
CH	0		5 (31.2%)			
CHRT	0		2 (12.5%)			
SUR	0		1 (6.2%)			
SUR + CH	0		1 (6.2%)			
SUR + CHRT	0		1 (6.2%)			
SUR + RT	0		1 (6.2%)			
RT	0		1 (6.2%)			
None	0		4 (25%)			
**Age diagnosis (years)**	**-**	42.88 ± 9.6	**-**	40.19 ± 7,7	0.39 95% CI (-8.99 - 3.61)	0.391***
**Age recurrence (years)**	-	44.25 ± 9.57	**-**	41.56 ± 7.4	0.38 95% CI (-8.87 - 3.49)	0.382***
**Recurrence**						
Endstage	1 (6.2%)		1 (6.2%)			**0.014***
LR	4 (25%)		12 (75%)			
Distance	11 (68.8%)		3 (18.8%)			
**Primary Chemotherapy**						
None	5 (31.2%)		11 (68.8%)			0.060*
Unknown	1 (6.2%)		2 (12.5%)			
Cisplatin	9 (56.2%)		2 (12.5%)			
Carboplatin/Taxol	0		1 (6.2%)			
Carboplatin/Cisplatin	1 (6.2%)		0			
**Recurrence Chemotherapy**						
none	13 (81.2%)		7 (43.8%)			0.081*
Cisplatin	0		2 (12.5%)			
Cisplatin/Topotecan	1 (6.2%)		0			
Carboplatin/Taxol	2 (12.5%)		2 (12.5%)			
Cisplatin/Taxol	0		1 (6.2%)			
Unknown	0		4 (25%)			
**Adjuvant Therapy**						
None	8 (50%)		6 (37.5%)			0.054*
RT	2 (12.5%)		6 (37.5%)			
CHRT	3 (18.8%)		1 (6.2%)			
SUR	3 (18.8%)		0			
HT	0		3 (18.8%)			
**Survival after recurrence (months)**		12.81 ± 9.4		11.44 ± 7.7	0.65 95% CI (-7.55 - 4.80)	0.587**
**Dead (number of patients)**	15 ((83.8%)		15 ((83.8%)			1.0*

Indicated p-values were generated via the Mann-Whitney non-parametric independent t-test, by Chi-Square test when indicated with a (*), by Wilcoxon Signed Rank test (**) or by parametric T-test (***).
